# pH-Responsive, Adorned Nanoniosomes for Codelivery of Cisplatin and Epirubicin: Synergistic Treatment of Breast Cancer

**DOI:** 10.1021/acsabm.1c01107

**Published:** 2022-02-07

**Authors:** Ali Moammeri, Koorosh Abbaspour, Alireza Zafarian, Elham Jamshidifar, Hamidreza Motasadizadeh, Farnaz Dabbagh Moghaddam, Zeinab Salehi, Pooyan Makvandi, Rassoul Dinarvand

**Affiliations:** † School of Chemical Engineering, College of Engineering, University of Tehran, Tehran 111554563, Iran; ‡ Faculty of Medicine, Isfahan University of Medical Sciences, Isfahan 8174673461, Iran; § Department of Pharmaceutical Nanotechnology, Faculty of Pharmacy, Tehran University of Medical Sciences, Tehran 141556451, Iran; ∥ Nanotechnology Research Center, Faculty of Pharmacy, Tehran University of Medical Sciences, Tehran 1316943551, Iran; ⊥ Department of Biology, Science and Research Branch, Islamic Azad University, Tehran 1477893855, Iran; # Istituto Italiano di Tecnologia, Center for Materials Interfaces, Pontedera, Pisa 56025, Italy

**Keywords:** breast cancer, cisplatin, epirubicin, endocytosis, folic acid, niosome

## Abstract

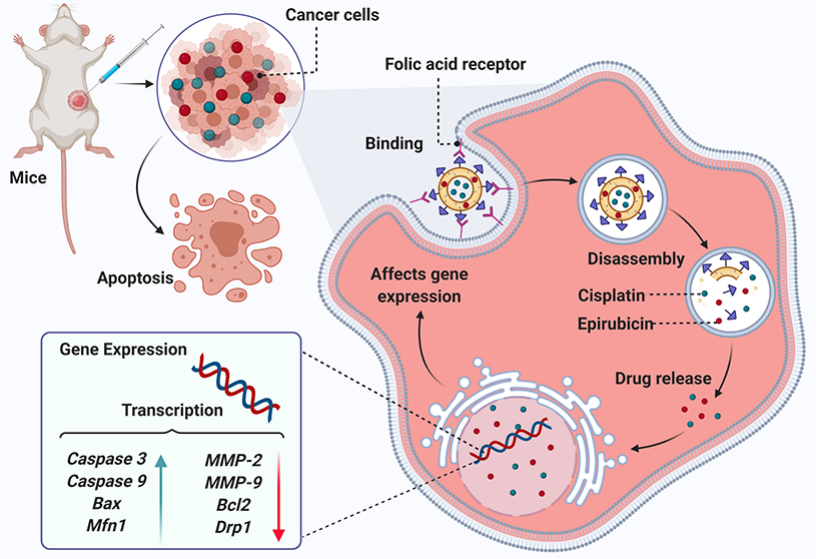

Combination chemotherapy has become a treatment modality for breast cancer. However, serious side effects and high cytotoxicity associated with this combination therapy make it a high-risk method for breast cancer treatment. This study evaluated the anticancer effect of decorated niosomal nanocarriers loaded with cisplatin (CIS) and epirubicin (EPI) *in vitro* (on SKBR3 and 4T1 breast cancer cells) and *in vivo* on BALB/c mice. For this purpose, polyethylene glycol (PEG) and folic acid (FA) were employed to prepare a functionalized niosomal system to improve endocytosis. FA-PEGylated niosomes exhibited desired encapsulation efficiencies of ∼91.2 and 71.9% for CIS and EPI, respectively. Moreover, cellular assays disclosed that a CIS and EPI-loaded niosome (NCE) and FA-PEGylated niosomal CIS and EPI (FPNCE) enhanced the apoptosis rate and cell migration in SKBR3 and 4T1 cells compared to CIS, EPI, and their combination (CIS+EPI). For FPNCE and NCE groups, the expression levels of *Bax*, *Caspase3*, *Caspase9*, and *Mfn1* genes increased, whereas the expression of *Bcl2*, *Drp1*, *MMP-2*, and *MMP-9* genes was downregulated. Histopathology results showed a reduction in the mitosis index, invasion, and pleomorphism in BALB/c inbred mice with NCE and FPNCE treatment. In this paper, for the first time, we report a niosomal nanocarrier functionalized with PEG and FA for codelivery of CIS and EPI to treat breast cancer. The results demonstrated that the codelivery of CIS and EPI through FA-PEGylated niosomes holds great potential for breast cancer treatment.

## 1 Introduction

Breast cancer is currently the most commonly diagnosed cancer in women (excluding nonmelanoma skin cancers).^1^ The main types of treatments for breast cancer are chemotherapy, surgery, endocrine (hormone) therapy (ET), radiation therapy (RT), and targeted therapy.^2^ Despite advances in cancer treatment strategies over the past few decades, chemotherapy is still considered as the main modality for cancer treatment. However, the use of chemotherapeutic drugs has been restricted because of some limitations, including multidrug resistance (MDR), insufficient efficacy, nonspecific biodistribution, and drastic side effects.^3^

Epirubicin (EPI) is a semisynthetic analog of doxorubicin (DOX) with reduced cardiac toxicity that is one of the most effective chemotherapy drugs in breast cancer treatment. EPI inhibits topoisomerase II and plays an important role in cancer therapy because it can inhibit DNA replication and lipid peroxidation.^4,5^ Cisplatin (CIS) is another effective chemotherapeutic drug that has been extensively used to treat human cancers such as breast, lung, neck, bladder, ovarian, head, and testicular types. CIS also causes DNA damage and induces apoptosis in cancer cells.^6^ However, cancer cell resistance against EPI remains a huge challenge for drug chemotherapy. This might be triggered by a reduction in intracellular EPI accumulation in cancer cells. Combination therapy with other anticancer agents, such as CIS and imatinib, offers EPI sensitivity improvement through the downregulation of the P-gp as a drug efflux pump and enhancement of the cellular uptake of EPI.^7^ Furthermore, CIS can be conjugated with carbonyl groups of polymer chains through a coordination bond,^8,9^ which is an effective strategy for combination with EPI.^10^ The good synergistic effect of these drugs against a wide range of cancer cell lines is due to the different mechanisms by which EPI and CIS act.^11−13^ Administration of CIS and EPI shows synergistic effects for breast cancer treatment by inducing several pathways contributing to cell apoptosis and metastatic behavior.^14,15^ Therefore, tumor-targeted codelivery of these two therapeutics using a highly biocompatible niosomal structure is a novel approach, which could increase our understanding of developing new nanocarriers in fighting the progression of cancer.^16^ Niosomes are biocompatible bilayer structures formed by the self-organization of nonionic surfactants and cholesterol that can overcome drawbacks associated with liposomes such as poor biocompatibility, low chemical stability, short storage life, and difficult large-scale production.^17,18^ Niosomes have gained much attention in drug delivery systems because of their unique properties, including exceptional biocompatibility, biodegradability, stability, nonimmunogenicity, and the ability to encapsulate both hydrophilic and hydrophobic drugs.^19−21^ Although vesicular systems like niosomes have special potential in cancer drug delivery, they still suffer from short blood circulation time and fast elimination by the reticuloendothelial system (RES). Surface decoration of niosomes with bioactive materials can effectively minimize the elimination by the RES, and therefore, the increased blood circulation improves and amplifies the endocytosis into cancer cells.^22,23^ Moreover, surface modification of niosomes with folic acid (FA), an active targeting ligand, enhances the folate receptor-mediated targeting delivery of nanoformulated drugs.^24,25^

In the present study, a FA-PEGylated nanoniosome was designed for the codelivery of both hydrophilic (CIS) and hydrophobic (EPI) chemotherapeutic drugs to treat breast cancer. To the best of our knowledge, this work describes the first combination therapy of CIS and EPI through FA-PEGylated niosomal nanocarriers for breast cancer therapy. For this purpose, CIS and EPI were loaded into a niosome (NCE), and then, it was decorated with FA and PEG (FPNCE). The release behavior of FA-PEGylated nanoniosomes was investigated in different pHs. The combined therapeutic efficacies of these functionalized niosomes were further evaluated in terms of cytotoxicity of the nanoformulations toward cancer and healthy cell lines. Real-time PCR and flow cytometry were used to evaluate the antiproliferative activity and mitochondrial dynamics involved in their mechanism of action. Furthermore, the migratory behavior of cells exposed to nanoformulations was assessed using scratch assay. Ultimately, the *in vivo* efficacy of the prepared nanoniosome was investigated using a 4T1 breast cancer model in BALB/c mice.

## 2 Materials and Methods

### 2.1 Preparation of Niosomal Formulations

The thin-layer hydration method was applied to prepare CIS and EPI-loaded niosomes.^26^ Briefly, Spans, cholesterol, and EPI were dissolved in 10 mL of chloroform/methanol (2:1; v/v) (according to Table S1). A rotary evaporator (150 rpm, 60 °C, 30 min) was used to evaporate the organic solvent (Heidolph Instruments, Germany). Then, the dried thin films were hydrated utilizing a CIS solution (in PBS, 10 mL, pH 7.4) at 60 °C for 30 min (120 rpm). Finally, the sample was sonicated for 7 min (Hielscher UP50H ultrasonic processor, Germany; amplitude, 25%, 200 W) to obtain the niosomal samples with uniform size distribution. The samples were stored in a refrigerator (4 °C) for further experiments. Eventually, to prepare the targeted formulation of FA-PEGylated nanoniosomes, 0.02 mol of FA-PEG-2000 was dissolved in 100 μL of methanol and added to the final niosome formulation containing CIS and EPI. The reaction was carried out for 2 h at 120 rpm and 45 °C in the rotary evaporator. After preparation, sonication was performed for 2 min in order to achieve uniform distribution of niosomes (amplitude, 25%, 200 W).

### 2.2 Determination of Encapsulation and Loading Efficiency (EE and LE)

The samples were centrifuged and ultrafiltered for 20 min at 4000*g* utilizing an Amicon. Throughout filtration, free drugs passed through the filter membrane, and the drug-loaded niosomes remained in the top chamber. The drug concentration at a wavelength of the maximum absorbance peak for CIS (360 nm) and EPI (480 nm) was analyzed by UV–visible spectroscopy (JASCO, V-530, Japan), and the drug concentration was evaluated according to its standard curve. Finally, EE and LE were measured using the following equations:
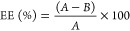
1
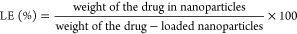
2

where “*A*” represents the amount of the initial drug trapped into the niosomal structures and “*B*” represents the non-niosomal-loaded drugs released from the membrane.

### 2.3 *In Vitro* Drug Release Kinetic Study

For the *in vitro* EPI and CIS release from niosomes, 2 mL of each niosomal sample and free drugs was added to a dialysis bag. A dialysis bag containing each sample was put in PBS-SDS (0.5%, w/v) solution (pH = 7.4 and 5.4) and stirred at 37 °C (50 rpm). Then, aliquots were taken at specified intervals and replaced with a fresh medium. Different kinetic models were employed to investigate and analyze the release profile.

### 2.4 Niosome Stability Studies

Stability was assessed by keeping the optimum formulation containing two drugs and the PEGylated formulation at 4 ± 1 °C (refrigeration temperature)/60% RH (relative humidity) ± 5% RH for 2 months; the physical properties in terms of the vesicle size (nm), PDI, and entrapment efficiency (%) were evaluated at certain time intervals (0, 30, and 60 days).

### 2.5 Cell Proliferation Assay

SKBR3 and 4T1 breast cancer cells were cultured in an RPMI 1640 medium (supplemented with 10% FBS, 100 IU/mL penicillin, 100 μg/mL streptomycin, and 2 mM l-glutamine). All cells were maintained under standard conditions for 24 h. Then, 12.5, 25, 50, 100, and 200 μg/mL concentrations of samples were added, and cells were incubated for 48 and 72 h. Cell proliferation was determined by cell viability assay. After treatment, the medium was replaced with a 0.5 mg/mL MTT solution and incubated for 4 h at 37 °C. Then, 100 μL of DMSO was added to dissolve the precipitated formazan, and the mixture was shaken for 20 min. A microplate reader was used to measure the absorbance (570 nm). Eventually, the IC_50_ was calculated for samples.

### 2.6 Calculation of the Combination Index

The combination index (CI) was studied to measure the combinatorial therapeutic effect resulting from the codelivery of CIS and EPI. CI > 1 implies antagonistic behavior, CI = 1 corresponds to additive behavior, and CI < 1 represents synergistic behavior. The CI was calculated based on the IC_50_ values obtained from the MTT assay by using the following formula (eq 3):
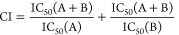
3where IC_50_(A) and IC_50_(B) are the IC_50_ values obtained from each drug separately. IC_50_(A + B) is the IC_50_ value of both drugs in combination.

### 2.7 Wound Healing Assay

SKBR3 and 4T1 cancer cell lines were seeded in 5 × 10^4^ cells/well and incubated until they reached 70% confluence to study cell migration. Then, a 200 μL pipette tip was used to scratch a monolayer of cells, and cells were then washed twice with PBS to remove floating cells. Cells were exposed to the IC_50_ samples in the medium for 72 h, then washed with PBS, and fixed, and microscopic photos were taken.

### 2.8 Flow Cytometric Analysis

To assess the apoptosis/necrosis ratio, the IC_50_ concentration was employed to treat SKBR3 and 4T1 cells for 72 h, and then, the annexin V/propidium iodide (PI) assay was used to study the cells, based on the manufacturer’s protocols. All cells were washed three times using cold PBS followed by resuspension in 1× binding buffer (5 × 10^5^ cells/well). Next, 5 μL of FITC annexin V and PI was added to 100 μL of each sample. The tubes were filled with 400 μL of 1× binding buffer. The cells without treatment were considered as the control group. The levels of apoptotic/necrotic cells were investigated by flow cytometry.

### 2.9 Gene Expression Analysis by Real-Time PCR

#### 2.9.1 RNA Extraction

Cells (1 × 10^7^) were seeded in 90 mm culture dishes and treated with IC_50_ of samples. Then, 1 mL of ice-cold RNX TM–Plus solution was added to a 2 mL tube containing the homogenized sample. Then, 200 μL of chloroform was added, the aqueous phase was transferred to a new RNase-free tube, and an equal volume of isopropanol was added. After centrifugation, the supernatant was discarded and washed with 1 mL of 75% ethanol, the pellet was dissolved in 50 μL of DEPC-treated water, and the total RNA was extracted based on the provided guidelines.

#### 2.9.2 cDNA Synthesis

Total RNA (1 ng–5 μg), Buffer-Mix (2×) (10 μL), Enzyme-Mix (2 μL), and DEPC-treated water were pipetted in an RNase-free tube for a total reaction volume of 20 μL. Then, the tube was incubated for 10 min at 25 °C and for 60 min at 47 °C. The reaction was discontinued by heating for 5 min at 85 °C. It was cooled on ice. The terminated RT reaction added up to 1.5 μL of the final PCR volumes for performing PCR.

#### 2.9.3 Primer Design and Real-Time PCR

The particular primers for *Drp1* (dynamin-related protein-1), *Mfn1* (*Mitofusin-1*)*, Bax* (Bcl-2-associated X protein), *Bcl2*, *Caspase3*, *Caspase9*, *MMP-2* (matrix metalloproteinase-2), *MMP-9* (matrix metalloproteinase-9), and β-actin (as an internal control) were designed through the National Center for Biotechnology Information (NCBI) website (Table S2).

#### 2.9.4 Quantitative Real-Time PCR

The following components were added: Real Plus and 2× Master Mix (12.5 μL vol/reaction, 1×), Primer A (10 μM) (0.5 μL (0.25–2.5 μL) vol/reaction, 0.1 μM (0.05–0.5 μM)), Primer B (10 μM) (0.5 μL (0.25–2.5 μL) vol/reaction, 0.1 μM (0.05–0.5 μM)), PCR-grade H_2_O (10.5 μL vol/reaction), and template DNA (1 μL), and the total volume was 25 μL (1 cycle, 15 min duration of the cycle, 95 °C; 40 cycles, 30 s duration of cycles at 95 °C and 30 s duration of cycles at 61.5 °C).

### 2.10 Cellular Uptake of Functionalized Niosomes

Qualitative cellular uptake of the formulation containing DOX was evaluated by confocal microscopy. First, niosomes containing DOX were prepared using the same preparation procedure as the EPI-loaded niosomes. Then, MCF-7 (human breast cancer cell line) cells were seeded in 6-well plates at a density of 5 × 10^4^ cells/mL for 24 h in DMEM (10% FBS, 100 IU/mL penicillin, and 100 μg/mL streptomycin). Then, niosomes loaded with DOX were added to each well. After 2 h, the treated cells were washed with PBS three times and fixed with paraformaldehyde (4%) for 5 min. Afterward, the cell nucleus was stained with Hoechst 33258 (2 μg/mL in each well). Finally, the cellular internalization of niosomes was observed by confocal microscopy (A1, Nikon, Switzerland).

### 2.11 *In Vivo* Study

#### 2.11.1 Experimental Animals and Ethical Aspects

All mice used in this study were kept under ethical considerations of the Institutional Animal Care and Use Committee of the Islamic Azad University, Science and Research Branch, Tehran, Iran (ethical code: 08/02/2021–614/8455). In this work, 25 female BALB/c inbred mice (weighing 18 ± 2 g, 6–8 weeks of age) were housed in animal polycarbonate cages (temperature of 22 ± 2 °C and humidity of 55%). After 7 days, the mice were randomly divided into 5 categories (*n* = 5 per group).

#### 2.11.2 Study Design

Intraperitoneal (i.p.) injection of the combination of CIS and EPI (CIS+EPI), NCE, and FPNCE was performed for 20 days (*n* = 5). Median lethal dose (LD_50_) values of the samples were considered in the treatment groups.

Group 1: cancer control, mice with breast cancer that received PBS.

Group 2: CIS+EPI.

Group 3: NCE.

Group 4: FPNCE.

#### 2.11.3 Induction of Breast Cancer

4T1 tumor cells (10^5^/mL in a suspension with phosphate-buffered saline, PBS 1×) were injected subcutaneously beneath the right side of the chest to induce the breast tumor. Between the 10th and 15th day, a solid tumor appeared subcutaneously. After 15 days, tumors were palpable.

#### 2.11.4 Histopathology

The tumor volume was measured using a digital caliper. Three mice from each group were sacrificed at the end of the treatment period. The tumor mass was removed and fixed in 10% buffered formalin for 48 h. Afterward, fixed tissues were embedded into paraffin blocks and sectioned into 5 μm-thick slices. The tumor sections were then stained with a hematoxylin and eosin (H&E) solution and microscopically examined at 400× magnification for histopathological features. Tumors were classified according to the Nottingham histologic score system (Menten grade) (Table 1). The amounts of gland formation, nuclear features, and mitotic activity were measured.

**Table 1:** Score of Histopathological Malignancy

index score	0	1	2	3
nuclear pleomorphism	no	small, regular nuclei	hyperchromatic, different sizes and shapes of nuclei	severe degree of the difference in nucleus size with hyperchromatic nuclei, one or more nuclei identified
invasion	absence in the dermis and hypodermis	presence in the dermis	infiltration into the hypodermis	penetration into the muscle tissue
mitosis	no	1–9	10–19	above 20

### 2.12 Statistical Analysis

Data were reported as the mean ± SD, and the graphs were plotted using OriginPro. Data were statistically analyzed using analysis of variances (ANOVA) followed by a post-Tukey test, and a *p* value less than 0.05 was considered as a significant difference.

## 3 Results

### 3.1 Preparation and Characterization

**Figure 1:**
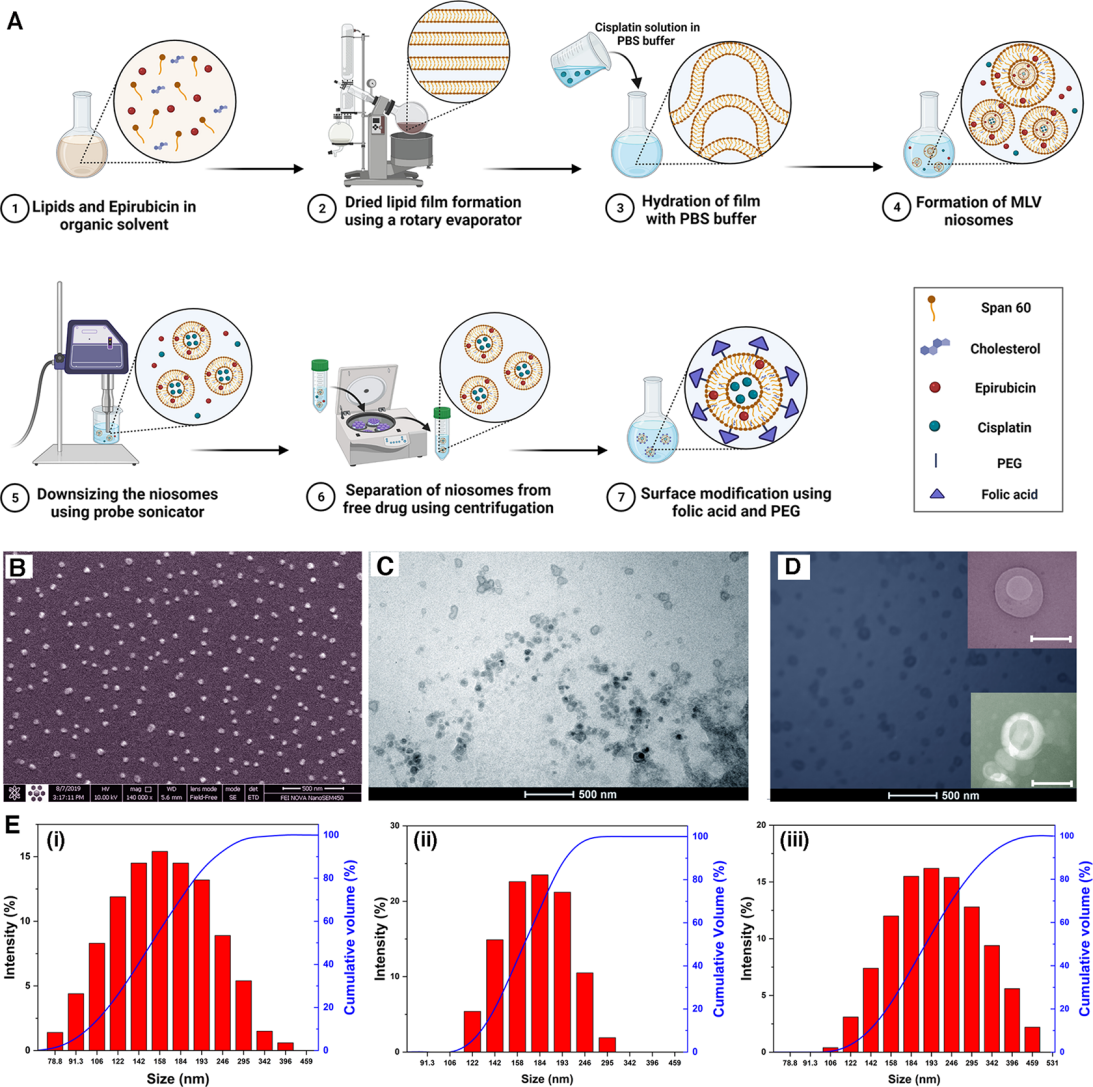
(A) Schematic illustration for the preparation of functionalized niosomes by a thin-layer hydration method. (B) Scanning electron microscopy (SEM) and (C) transmission electron microscopy (TEM) images of NCE5; the scale bar represents 500 nm. (D) TEM images of FA-PEGylated niosomes (FPNCE). Scale bars of the magnified images represent 100 and 275 nm for upside and downside panels, respectively. (E) Analysis of particle size distribution of empty niosome (i), NCE (ii), and FPNCE (iii).

Three important parameters, i.e., the size, PDI, and EE, were observed for eight formulations, all loaded with 10 mg of each drug, using three different surfactants (Span 20, Span 60, and Span 80) (Table S1). First, Span 20 was combined with cholesterol at a 1:1 ratio (200 μmol of lipids), then an exact ratio and lipid volume for Span 60, and finally for Span 80. Span 80 was the most suitable, as it showed an acceptable size, PDI, and EE; because of the liquid characteristic of Span 80, however, it is less applicable as a delivery vesicle (Table S1). Aiming for a higher EE, a 1:2 ratio of surfactant/cholesterol was added to 300 μmol of lipids (Figure 1A). As the results indicate, all nanoparticle sizes increased due to the higher lipid volume, and the EE improved because of a thicker lipid layer; with increasing the cholesterol amount, a higher EE was obtained.^27,28^

Span 60 resulted in the optimum formulation. Span 60 was chosen to prepare FA-PEGlyated niosomes due to its organogel characterization, making it a suitable delivery vesicle. Surprisingly, Span 60 showed an enhanced EE and PDI improvement, while it produced smaller sizes than Span 20 or Span 80. Therefore, NCE5 was chosen as the optimized formulation. It can be concluded from the results shown in Table S1 that the PEGylated formulation showed a higher EE and PDI with just a slight size expansion, indicating that FA-PEGylation resulted in a small increase in niosome size with a better PDI and an improved EE.

The morphology of the NCE was characterized using SEM and TEM techniques (Figure 1B–D). As can be seen, the carriers demonstrated a smooth surface, a spherical shape, and separated firm boundaries, with a homogeneous distribution. Nanoparticle size distribution was assessed using DLS, showing that empty niosomes possess an average diameter of ∼158 nm (“i” in Figure 1E). The nonfunctionalized niosomes (NCE samples) increased by about 30 nm and reached to ∼184 nm in size (“ii” in Figure 1E), whereas the size of the functionalized samples showed ∼192 nm (“iii” in Figure 1E). The size of niosomes obtained by TEM was smaller than that obtained by DLS data. In agreement with other literature studies, the difference in size measurement between TEM and DLS methods is due to the fundamental difference between intensity and number-weighted particle size distributions and the differences between the dry and hydrodynamic radius of particles.^29−31^

**Figure 2:**
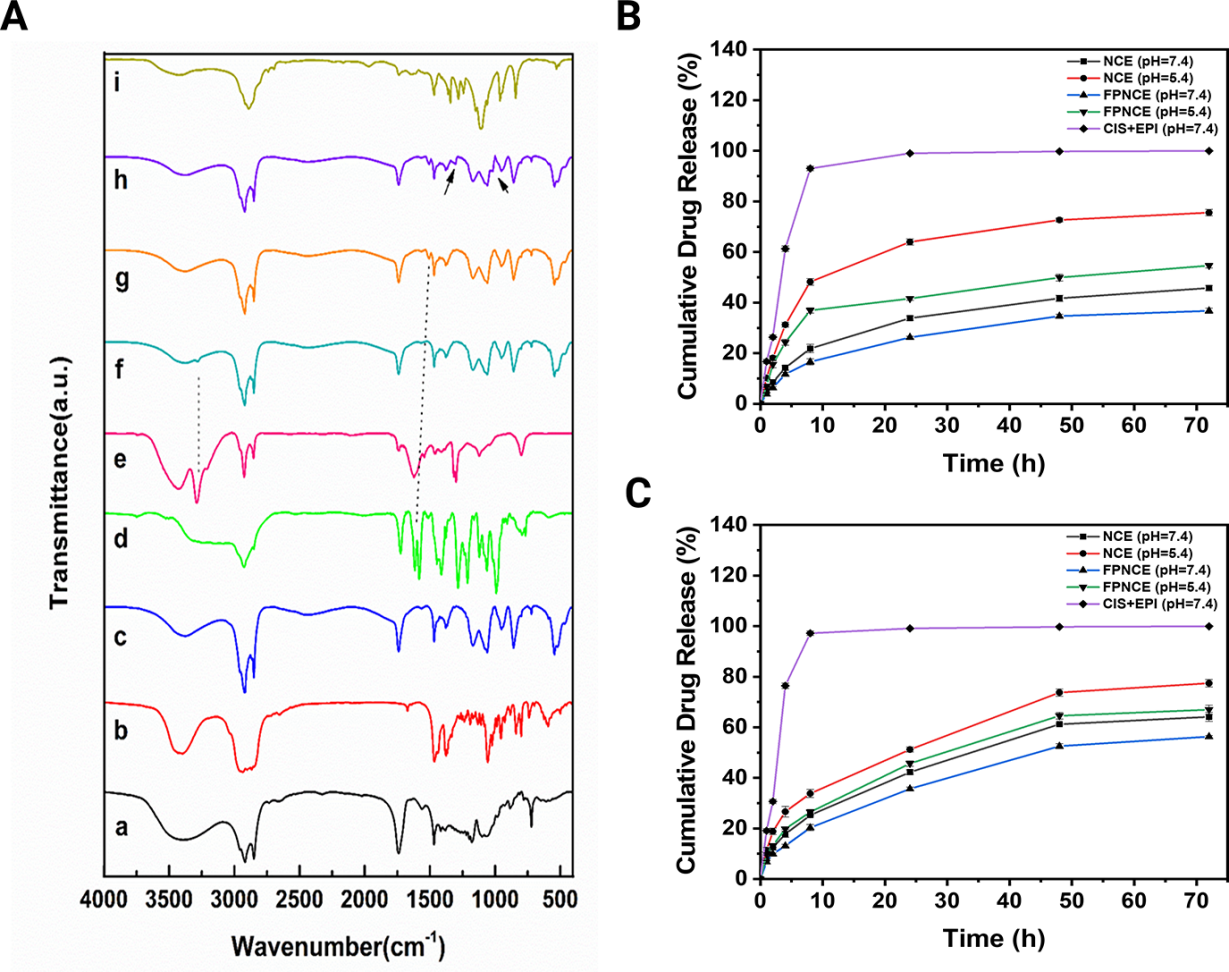
(A) FT-IR spectra of (a) Span 60, (b) cholesterol, (c) empty niosome, (d) EPI, (e) CIS, (f) CIS*-*loaded niosome, (g) EPI*-*loaded niosome, (h) FA-PEGylated niosomes, and (i) FA-PEG-2000. (B) *In vitro* release of CIS and (C) EPI from a dialysis bag containing the drug solution, NCE, and FPNCE at different pHs (7.4 and 5.4). Data are shown as the mean ± SD, *n* = 3.

An FT-IR study was conducted to detect and demonstrate the presence of and interactions between components (Figure 2A). The components were analyzed individually and in combination. In the following paragraph, each figure’s diagram is explained. For Span 60 in Figure 2A (“a”), the wavenumbers of 1250, 2800–3000, and 3452 cm^–1^ are noticeable, which indicate C–O, C–H, and O–H stretching, respectively. For cholesterol in Figure 2A (“b”), the wavenumbers of 2800–3000, 3452, 1035–1378, 1506, and 1674 cm^–1^ are noticeable, which relate to C–H, O–H, CH_2_ bending and CH_2_ deformation, the C–C aromatic ring, and C=C, respectively. As shown in the Figure 2A (“c”) spectrum (the empty niosomes), both wavenumbers 1125 and 1747 cm^–1^ represent the combination of Span 60 and cholesterol, which was the approach used in this study to formulate the niosomes. Figure 2A (“d”), free EPI FT-IR, shows wavenumbers of 2918, 1720, and 1400–1600 cm^–1^ relating to C–H, C=O, and the C=C aromatic ring, respectively. For CIS in Figure 2A (“e”), wavenumbers of 3285, 1303, and 749 cm^–1^ belong to amine stretching, symmetric amine bending, and chloride stretching, respectively. In the next step, the drugs were loaded into the niosomes; in the Figure 2A (“f”) spectrum, CIS was added to niosomes, and the wavenumber of 3285 cm^–1^ (amine stretching) was an assurance that loading was successful; then, the Figure 2A (“g”) spectrum shows the 1505 cm^–1^ C=C aromatic wavenumber, proving that EPI was loaded in the empty niosomes. Subsequently, the Figure 2A (“h”) spectrum refers to the final formulation (FPNCE). As PEG and FA were added to the system, CIS’ wavenumbers disappeared, and PEG and FA wavenumbers increased to 1307 cm^–1^ for the C–H bond in PEG and 1020 cm^–1^ for the C–N bond in FA (wavenumbers extracted from the Figure 2A (“i”) spectrum, relating to PEG and FA separately).

### 3.2 *In Vitro* Drug Release and Kinetic Studies

To investigate the *in vitro* drug release, each optimum formulation drug release profile was observed for 72 h in 7.4 and 5.4 pH at body temperature. According to Figure 2B,C, free drugs first had burst for 8 h and were then released monotonously for the remaining 16, 40, and 64 h. The CIS release profile showed that 45.75% of the drug had penetrated the target in 7.4 pH; this rate increased to 75.55% in 5.4 pH because the acidic condition swelled the niosome structure.^32^ EPI release surveillance indicated that 64.11% of the drug entered the target in 7.4 pH, and the entrance rate increased to 77.39% in 5.4 pH, again due to destruction of the acidic conditions. FPNCE samples were examined, and the CIS release percentage in the FA-PEGylated formulation was found to be 36.78% in 7.4 pH and 56.30% for EPI; both of these rates increased to 54.63 and 66.93% in 5.4 pH, respectively. These results showed that FA-PEGylation hindered drug release, which caused more drug accumulation in the target cells, and acidic pH broke the niosome structure, subsequently increasing the released rate, which can increase the toxicity as the acidic condition in the tumor regions.^33^ The release data of EPI and CIS were mathematically calculated in the zero order, first order, Korsmeyer–Peppas order, and Higuchi’s order in two pH values (7.4 and 5.4) at body temperature (Table S3). Free drug release followed the first-order model, which represented drug-dependent concentration (this applies for both EPI and CIS as separate free drugs). Both EPI-loaded and CIS-loaded niosomes followed the Korsmeyer–Peppas model in both 7.4 and 5.4 pH. This fact indicated that the release mechanism is the diffusion–erosion composition. The CIS release profile in the final formulation (FPNCE) fitted Higuchi’s model in both 7.4 and 5.4 pH with determination coefficients (*R*^2^) of 0.9684 and 0.8770, respectively, indicating the diffusion coefficient release model. However, the final EPI release profile obeyed the Korsmeyer–Peppas model in both 7.4 and 5.4 pH.^34^

### 3.3 Physical Stability Study

To examine the optimal FPNCE formulations and physical stability, the vesicle size, PDI, and EE were investigated on days 0, 30, and 60 after preparation at 4 °C. As Table 2 shows, PEGylated formulations revealed better stabilities than NCE formulation. According to Table 2, an increase in the particle size and PDI together with a drop in the EE of NCE particles compared with FPNCE suggested that FA-PEGylated formulations exhibited more stability than nonfunctionalized niosomes. In comparison with the NCE, PEGylated formulations displayed less significant variation in terms of stability characteristics, including the particle size, PDI, and EE. In other words, the increase rate in the vesicle size and PDI together with the decrease rate in EE for FPNCE is lower than those for the NCE during the storage time. Hence, the results indicate that FA-PEGylation stabilized the formulation because the polymerized niosomes reduced the systematic phagocytosis.^35^

**Table 2:** Stability of Optimum NCE and FPNCE Formulations Stored during 2 Months of Storage at 4 ± 2 °C^a^

	vesicle size (nm)			PDI		
time of storage (day)	NCE	FPNCE	*p* value	NCE	FPNCE	*p* value
0	184.0 ± 4.5	192.5 ± 8.9	NS	0.103 ± 0.007	0.142 ± 0.012	<0.05
30	231.7 ± 9.5	224.5 ± 11.7	NS	0.143 ± 0.014	0.175 ± 0.024	<0.05
60	297.9 ± 13.2	275.6 ± 14.5	NS	0.274 ± 0.024	0.235 ± 0.017	<0.01

	EE_(CIS)_ (%)			EE_(EPI)_ (%)		
time of storage (day)	NCE	FPNCE	*p* value	NCE	FPNCE	*p* value
0	85.48 ± 1.23	91.24 ± 1.32	<0.01	68.52 ± 1.48	71.93 ± 1.11	NS
30	80.37 ± 1.75	89.75 ± 0.67	<0.01	63.49 ± 2.08	69.25 ± 1.25	<0.01
60	76.21 ± 1.78	85.29 ± 1.45	<0.01	59.54 ± 0.91	65.28 ± 2.24	<0.01

a Data presented as the average ± SD, *n* = 3; NS: not significant.

### 3.4 Cell Proliferation Assay

**Figure 3:**
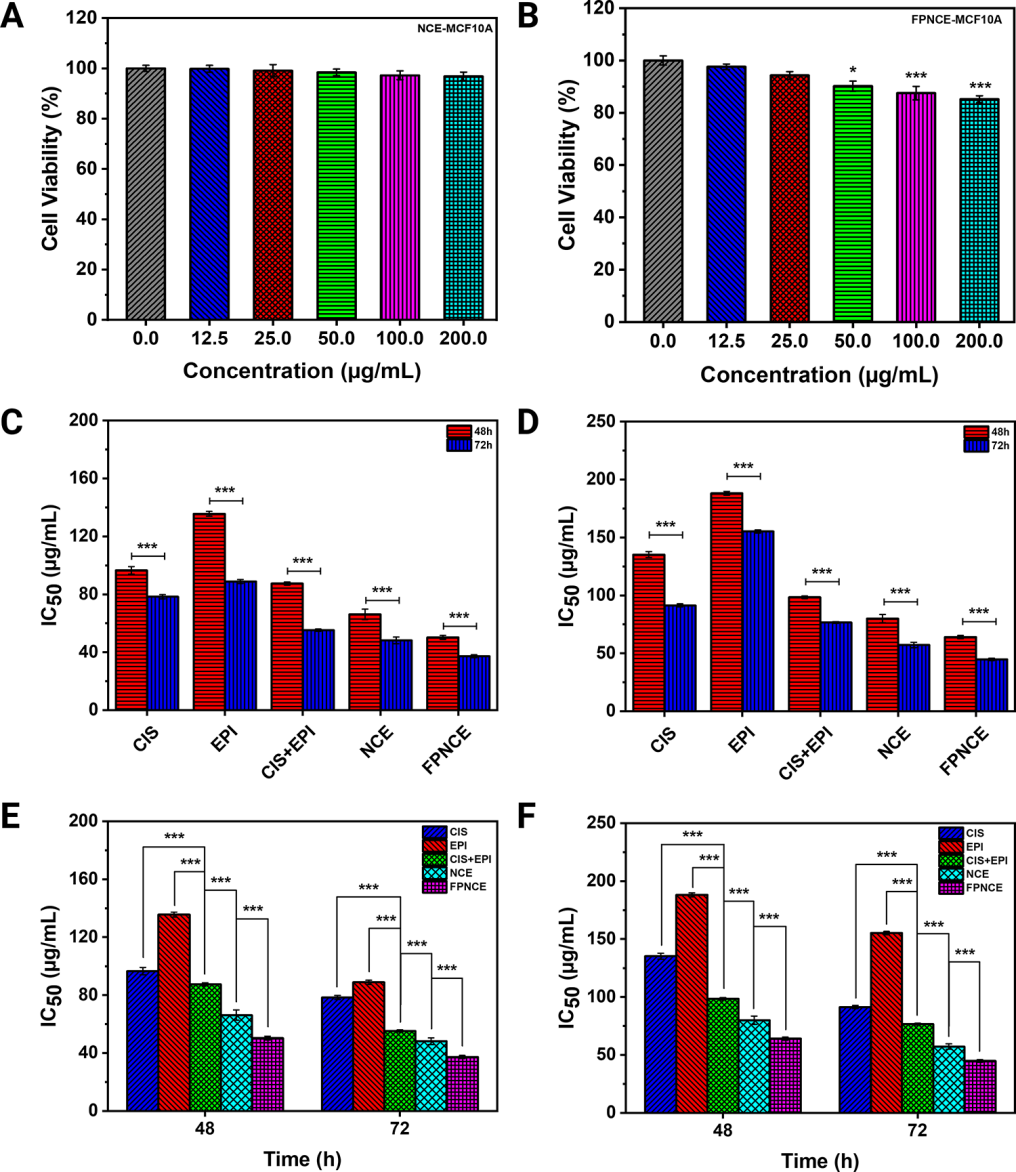
(A) Cell viability of NCE concentration on a healthy MCF10A cell line. (B) Cell viability of FPNCE dilution on a healthy MCF10A cell line. IC_50_ values in CIS, EPI, CIS+EPI, NCE, and FPNCE (*p* < 0.001) on (C) 4T1 cells and(D) SKBR3 cells after 48 and 72 h. CIS, EPI, CIS+EPI, NCE, and FPNCE IC_50_ values compared with each other after 48 and 72 h on (E) 4T1 cells and (F) SKBR3 cells. Data are shown as the mean ± SD, *n* = 3. The mean values with asterisks are significantly different (*p* ≤ 0.05).

MTT assay was performed to investigate the *in vitro* performance of CIS, EPI, CIS+EPI, NCE, and FPNCE on SKBR3 and 4T1 cells. First, the effects of NCE and FPNCE samples on the viability of MCF10A cells (nonmalignant breast epithelial cells) were investigated. The MCF10A cells were exposed to different concentrations of niosomes, which showed no statistically significant changes in the percentage of cell viability (Figure 3A). The investigation of FPNCE’s effect on MCF10A cells revealed that in concentrations of 50, 100, and 200 μg/mL, significant decreases in the percentage of cell viability were observed (*p <* 0.05, *p <* 0.001, and *p <* 0.001, respectively; Figure 3B).

The half-maximal inhibitory concentration (IC_50_) values were evaluated in all study groups on 4T1 cells 48 and 72 h after treatment. The IC_50_ values of different samples were 96.56 ± 2.57 μg/mL for CIS, 135.63 ± 1.68 μg/mL for EPI, 87.47 ± 1.10 μg/mL for CIS+EPI, 66.19 ± 3.57 μg/mL for NCE, and 50.30 ± 1.35 μg/mL for FPNCE after 48 h (Figure 3C) and 78.35 ± 1.42 μg/mL for CIS, 88.85 ± 1.39 μg/mL for EPI, 55.24 ± 0.76 μg/mL for CIS+EPI, 48.18 ± 2.33 μg/mL for NCE, and 37.27 ± 1.07 μg/mL for FPNCE after 72 h (Figure 3C). As expected, the cytotoxic effect increased after 72 h compared to 48 h for all study groups because there were statistically significant decreases in the IC_50_ values of CIS, EPI, CIS+EPI, NCE, and FPNCE after 72 h (*p <* 0.001 for all comparisons; Figure 3C,D). In addition, compared with CIS and EPI, there was a statistically significant decrease in the IC_50_ value of CIS+EPI (*p <* 0.001 for all comparisons; Figure 3E). The IC_50_ values for niosomal formulations, especially those functionalized with PEG and FA (FPNCE), were also significantly lower than those of other samples (*p <* 0.001 for all comparisons; Figure 3E).

Similarly, the IC_50_ values were evaluated in all study groups on SKBR3 cells 48 and 72 h after treatment. The IC_50_ values of different samples were 135.20 ± 2.57 μg/mL for CIS, 188.16 ± 1.68 μg/mL for EPI, 98.43 ± 1.10 μg/mL for CIS+EPI, 79.96 ± 3.57 μg/mL for NCE, and 64.08 ± 1.35 μg/mL for FPNCE after 48 h (Figure 3D) and 91.35 ± 1.42 μg/mL for CIS, 155.21 ± 1.39 μg/mL for EPI, 76.63 ± 0.76 μg/mL for CIS+EPI, 57.24 ± 2.33 μg/mL for NCE, and 44.82 ± 1.07 μg/mL for FPNCE after 72 h (Figure 3D). As expected, the cytotoxic effect increased after 72 h compared to 48 h for all study groups because there were statistically significant decreases in the IC_50_ values of CIS, EPI, CIS+EPI, NCE, and FPNCE after 72 h (*p <* 0.001 for all comparisons; Figure 3C,D). In addition, compared with CIS and EPI, there were statistically significant decreases in the IC_50_ values of CIS+EPI (*p <* 0.001 for all comparisons; Figure 3F). The IC_50_ values for niosomal formulations, especially those for FPNCE, were also significantly lower than those of other samples (*p <* 0.001 for all comparisons; Figure 3F).

### 3.5 Combination Index Analysis

According to the IC_50_ values of the studied groups on 4T1 and SKBR3 cancer cells, the synergistic effects of CIS and EPI were tested using the Chou–Talalay combination index equation (eq 3).^36^ After 72 h in 4T1 cells, the CI values of the CIS+EPI and NCE were 1.3 and 1.1, respectively, which do not confirm the synergism of the two drugs. On the contrary, the FPNCE was 0.8, indicating the synergistic activity. Similarly, in the SKBR3 cell line, the CI of CIS+EPI was 1.29, showing no synergistic effect for CIS and EPI. In contrast, the NCE exhibited synergism (CI = 0.9). In addition, the combination of drugs incorporated into FA-PEGylated niosomes enhanced the synergistic activity of CIS and EPI in the encapsulated form (CI = 0.7). The results demonstrated that codelivery without a nanocarrier exhibited no synergistic effect, whereas the encapsulated form of the drugs displayed a synergistic activity.

### 3.6 Scratch Assay

**Figure 4:**
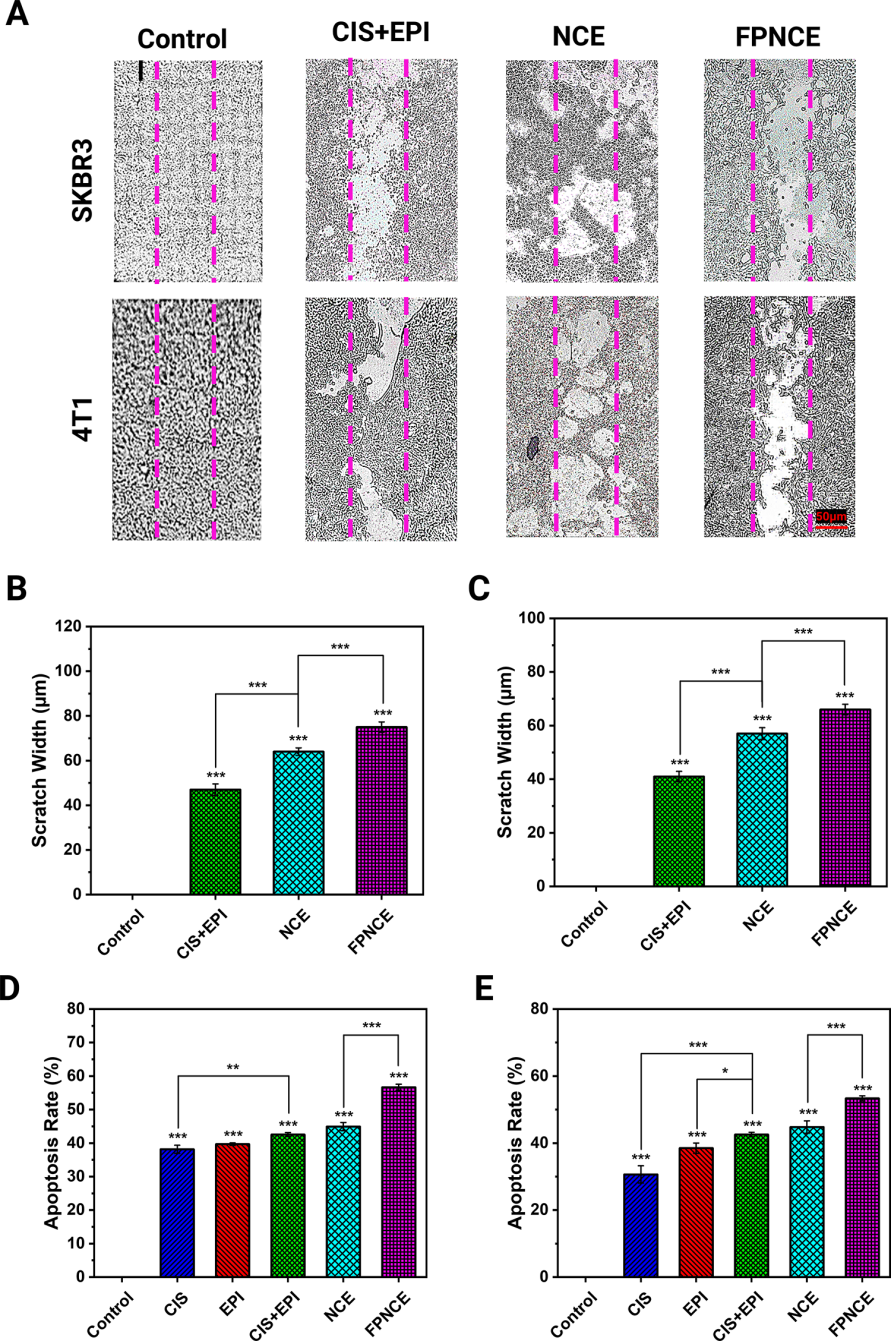
(A) Macroscopy inhibitory effects of CIS+EPI, NCE, and FPNCE on the migration and invasion of the SKBR3 and 4T1 breast cancer cells after 72 h of treatment. Inhibitory effects of CIS+EPI, NCE, and FPNCE on the migration and invasion of (B) 4T1 and(C) SKBR3 breast cancer cells after 72 h of treatment. Apoptosis assay by FITC and PI using flow cytometry on (D) 4T1 and (E) SKBR3 cells treated with CIS, EPI, CIS+EPI, NCE, and FPNCE. Data are shown as the mean ± SD, *n* = 3. The mean values with asterisks are significantly different (*p* ≤ 0.05).

**Figure 5:**
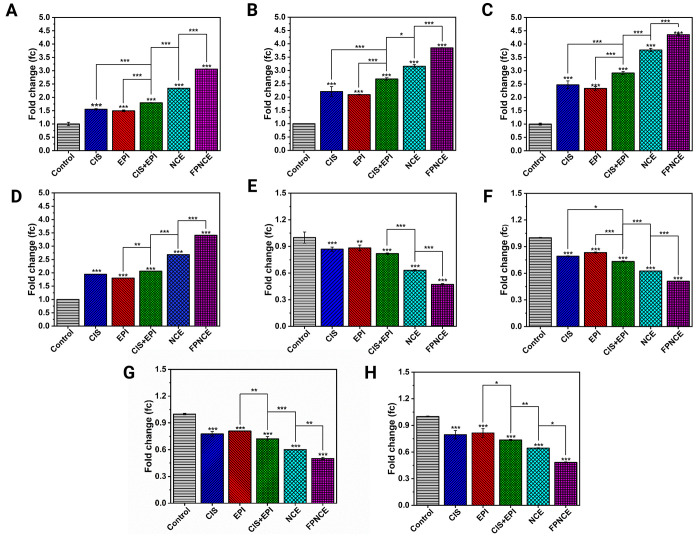
Expression levels of (A) *Bax*, (B) *Caspase3*, (C) *Caspase9*, (D) *Mfn1*, (E) *Bcl2*, (F) *Drp1*, (G) *MMP-2*, and (H) *MMP-9* in 4T1 cells after being treated with CIS, EPI, CIS+EPI, NCE, and FPNCE. Data are shown as the mean ± SD, *n* = 3. The mean values with asterisks are significantly different (*p* ≤ 0.05).

To determine the effect of CIS+EPI, NCE, and FPNCE on migration and invasion, the cell scratch test (4T1 and SKBR3) was used for 72 h. FPNCE had more migration inhibitory effects than the other groups; however, this effect was minimal only with the use of CIS and EPI. In addition, microscopic images showed the antimigratory and invasive effects of CIS+EPI, NCE, and FPNCE on SKBR3 and 4T1 cells after 72 h (Figure 4A). As shown in Figure 4B,C, the scratch width (μm) of the SKBR3 and 4T1 cell lines treated with FPNCE was higher than those of the NCE and CIS+EPI groups. The scratch width of 4T1 cells was increased after being treated with CIS+EPI, NCE, and FPNCE compared to the control (*p* < 0.001) (Figure 5B). To be more specific, niosomal formulations showed that FPNCE had the greatest inhibitory effect relative to the non-niosomal formulation on 4T1 cells. Likewise, NCE, CIS+EPI, and FPNCE treatment groups reduced SKBR3 cell migration and enlarged the scratch width (μm) compared to the untreated group (*p* < 0.001) (Figure 5C). In both cell lines, the FPNCE-treated group showed a statistically significant increase in the scratch width compared to the NCE (*p* < 0.001) and the NCE compared to CIS+EPI (*p* < 0.001). According to microscopic images of SKBR3 and 4T1 cells after 72 h of treatment, it was observed that FPNCE prevented the migration and division of SKBR3and 4T1 cells to a higher extent than the NCE (*p* < 0.001). Thus, the scratch width of FPNCE, NCE, compared to the control, and CIS+EPI showed the effectiveness of this drug delivery system in inhibiting breast cancer cell migration and ultimately controlling the tumor, which prevents it from invasion.

### 3.7 Flow Cytometry Analysis

The apoptosis effects of CIS, EPI, CIS+EPI, NCE, and FPNCE were provided in 4T1 and SKBR3 cells (Figure 4D,E). Flow cytometric analysis indicated a significant induction in apoptosis percentage in 4T1 and SKBR3 cells after treatment with CIS, EPI, CIS+EPI, NCE, and FPNCE IC_50_ compared to the control.

The results demonstrate the apoptosis rate of cancer cells exposed to free drugs, CIS+EPI, NCE, and FPNCE. In the case of the 4T1 cell line, the results were 38.14, 39.67, 42.54, 44.93, and 56.66% (Figure 4D) and, in the same way, 30.63, 38.48, 42.54, 44.77, and 53.33% for SKBR3 cells (Figure 4E), which indicate increasing apoptosis in SKBR3 and 4T1 cells. These results are in agreement with the cytotoxicity data obtained by MTT assay. According to Figure 4D, the apoptotic rate (%) of FPNCE was higher than that of the NCE, the CIS+EPI was higher than CIS, and apoptotic rates in all groups with treatment with CIS, EPI, CIS+EPI, NCE, and FPNCE were higher compared to the control group in the 4T1 cell line. Additionally, the apoptotic rate (%) on SKBR3 showed that FPNCE was higher than the NCE, and CIS+EPI was higher than CIS and EPI. It is also important to note that the apoptotic rate in all groups treated with CIS, EPI, CIS+EPI, NCE, and FPNCE showed an increase in apoptotic activity compared to the control in the SKBR3 cell line (Figure 4E). The data suggest that all groups led to cell apoptosis; however, only FPNCE appeared to be in line with the IC_50_ results.

Following confirmation that the FPNCE and NCE have cytotoxicity effects on SKBR3 and 4T1 cells, the percentage of apoptosis was assessed for all groups. FPNCE induced apoptosis in SKBR3 and 4T1 cells, and this percentage showed a significant increase compared to the NCE, EPI, and CIS groups.

### 3.8 Real-Time PCR

**Figure 6:**
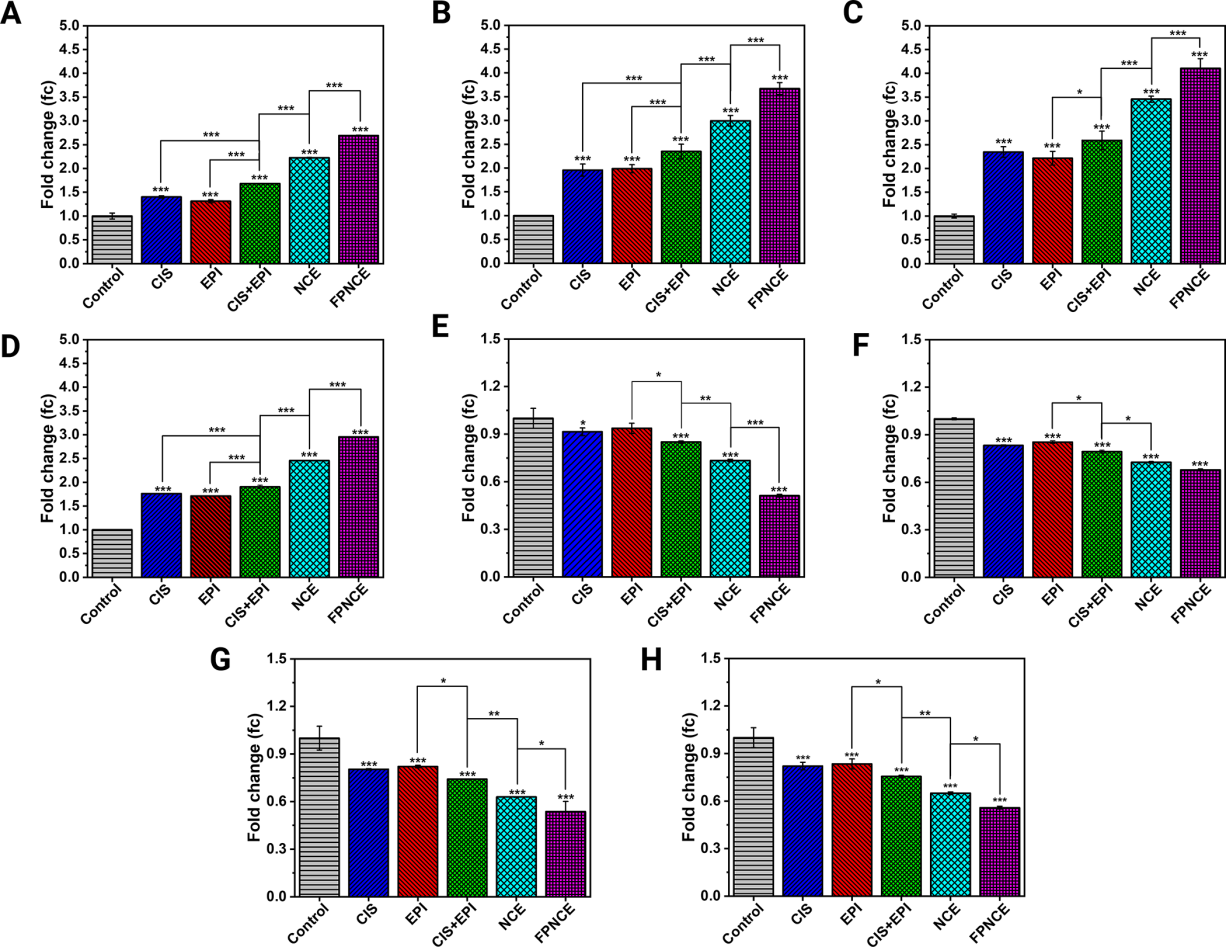
Expression levels of (A) *Bax*, (B) *Caspase3*, (C) *Caspase9*, (D) *Mfn1*, (E) *Bcl2*, (F) *Drp1*, (G) *MMP-2*, and (H) *MMP-9* in SKBR3 cells after being treated with CIS, EPI, CIS+EPI, NCE, and FPNCE. Data are shown as the mean ± SD, *n* = 3. The mean values with asterisks are significantly different (*p* ≤ 0.05).

The gene expression levels in the 4T1 and SKBR3 cell lines were measured quantitatively by real-time PCR to examine the effectiveness of different niosomal formulations (CIS, EPI, CIS+EPI, NCE, and FPNCE). The eight genes investigated were *Bax*, *Bcl2*, *Caspase3*, *Caspase9*, *Mfn1*, *Drp1*, *MMP-2*, and *MMP-9* in 4T1 (Figure 5 A–H) and SKBR3 (Figure 6 A–H). All niosome groups had upregulated expression levels of *Bax*, *Caspase3*, *Caspase9*, and *Mfn1* compared to the control (*p <* 0.001) in both 4T1 and SKBR3 cells. The NCE group increased *Bax*, *Caspase9*, and *Mfn1* expression levels significantly in both cells compared with the CIS+EPI group (*p <* 0.001). Similarly, the NCE had a greater effect on the *Caspase3* expression level than CIS+EPI in 4T1 cells (*p* < 0.05; Figure 5B). In addition, in 4T1 and SKBR3 cells, the expression levels of *Bax*, *Caspase3*, *Caspase9*, and *Mfn1* genes in cells exposed to FPNCE were remarkably higher than in cells exposed to the NCE (*p* < 0.001) (Figures 5A–D and 6A–D). For *Caspase9* expression, the CIS+EPI group showed a higher increase than EPI (*p* < 0.05; Figure 6C) in the SKBR3 cell line, and the *Mfn1* expression level in 4T1 cells for CIS+EPI was higher than that for EPI (*p* < 0.01; Figure 5D).

All experimental niosome groups had downregulated expression levels of *Bcl2*, *Drp1*, *MMP-2*, and *MMP-9* in both the 4T1 and SKBR3 cancer cells compared to the control (*p <* 0.001; Figures 5E–H and 6E–H). For *Bcl2*, the FPNCE-treated group had less gene expression than the NCE (*p* < 0.001)-treated group. Moreover, NCE treatment exposed less expression than CIS+EPI treatment in the 4T1 and SKBR3 cell lines (*p <* 0.001 and *p <* 0.01, respectively). The lower levels of *Drp1* gene expression in 4T1 and SKBR3 cells, according to Figures 5F and 6F, correspond to higher cell denaturation, suggesting FPNCE transcendent efficacy (51% of the control group in 4T1 and 67% of the control group in SKBR3). The expression levels of *MMP-2* and *MMP-9* in FPNCE were lower than those in the NCE (*p* < 0.05); the expression levels in the NCE in both cancer cell lines were the same and showed lower amounts compared to CIS+EPI (*p* < 0.01).

In summary, *Bax*, *Caspase3*, *Caspase9*, and *Mfn1* were upregulated in both cell lines after their exposure to all niosome groups (*p <* 0.001). Conversely, *Bcl2*, *Drp1*, *MMP-2*, and *MMP-9* were downregulated in both cells after their exposure to all niosome formulations (*p <* 0.001). These fold differences between groups were the highest in the FPNCE experimental group.

### 3.9 Cellular Uptake of Functionalized Niosomes

**Figure 7:**
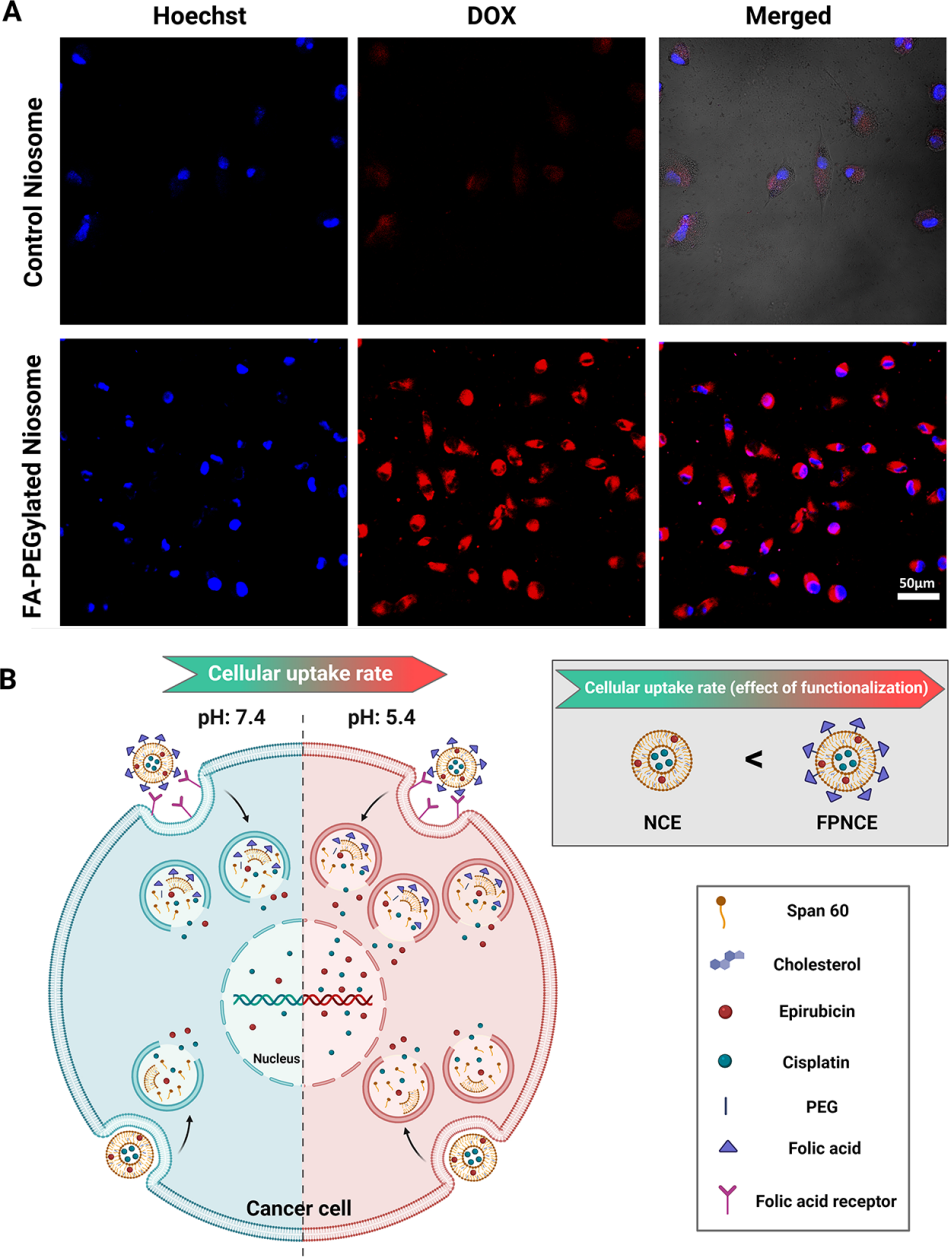
(A) Fluorescence images of MCF7 cells after 2 h of incubation with the control niosome and the FA-PEGylated niosome. The scale bar represents 50 μm. Note: cells were stained with Hoechst 33258 for visualization of cell nuclei (blue), and DOX was used for niosome tracing (red, pseudocolor). (B) Schematic representing the effect of pH on the release of contents from a niosome.

To evaluate cellular uptake of modified niosomes, MCF7 cells were stained with DOX, a red fluorescent anticancer model drug. Cellular uptake confirmed the internalization of dual drug-loaded niosomes at 37 °C. Cell nuclei were stained with Hoechst 33258 and showed blue, and DOX showed red. Figure 7A reveals that the fluorescence strength of the cells was very weak when the cells were incubated with drug-loaded niosomes. In contrast, Figure 7A shows higher internalization of drugs by FA-PEGylated niosomes. Therefore, the results regarding cellular uptake revealed that the modification of niosomes with FA facilitates the modified niosome accumulation in MCF7 cells, which means that the cytotoxic effect on cancerous cells was enhanced after treatment with targeting niosomes. The most profuse cell uptake happened in MCF7 cells treated with FPNCE. As depicted in Figure 7B, the release rate of CIS and EPI from FA-decorated niosomes at pH 5.4 was higher than that of the free form of drugs and the release rate at pH 7.4. Moreover, endocytosis was prominent in the cells treated with FPNCE in the sample group compared with MCF7 cells exposed to the NCE group.

### 3.10 Histopathology

**Figure 8:**
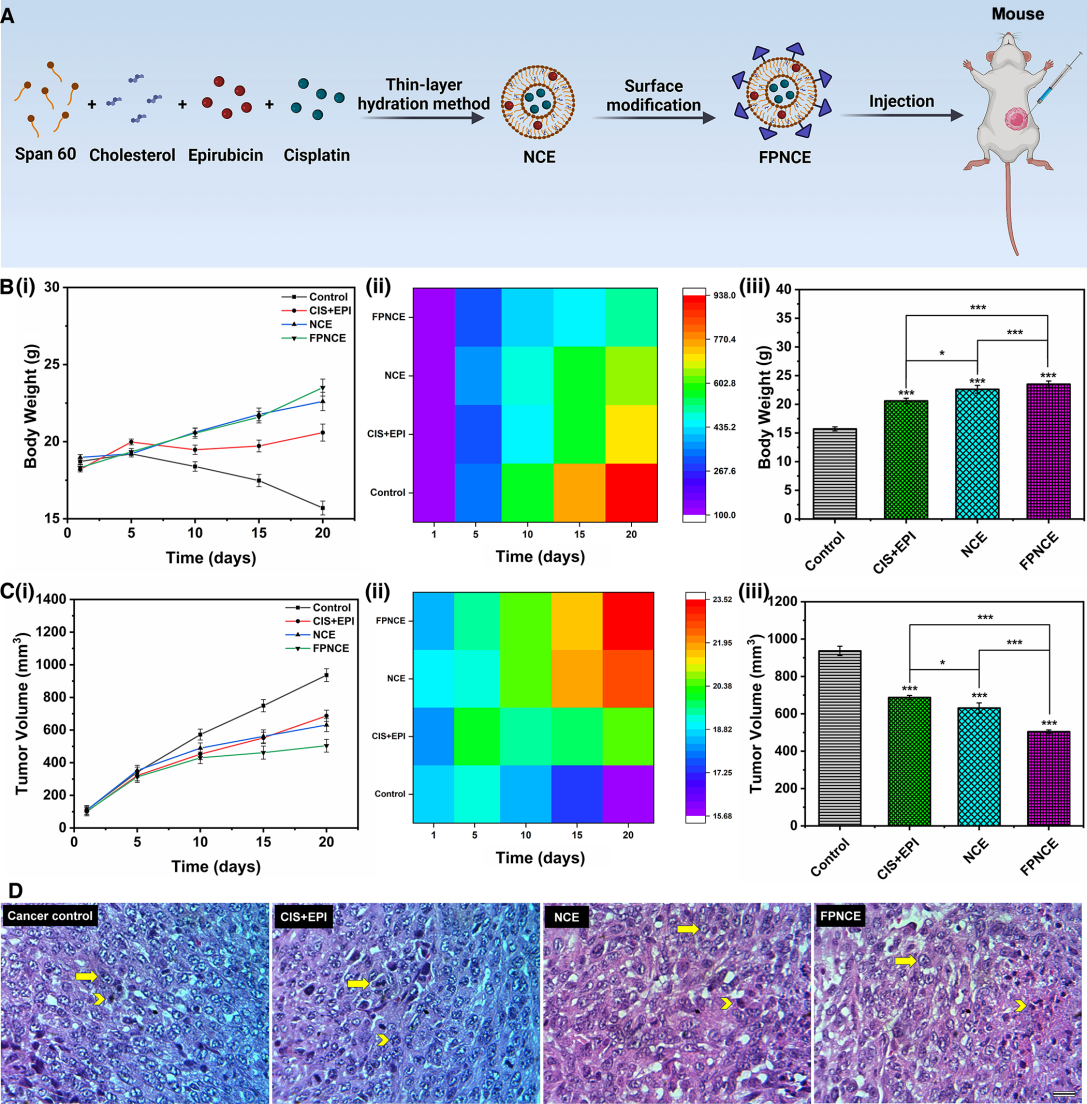
(A) Schematic illustration on codelivery of CIS+EPI to a mouse. (B) Mouse body weight against time (i) and heat map of mouse body weight (ii). Mouse body weight on the 20th day of treatment (iii). (C) Tumor volume against time (i) and heat map of the tumor volume (ii). Tumor volume on the 20th day of treatment (iii). Data are shown as the mean ± SD, *n* = 3. The mean values with asterisks are significantly different (*p* ≤ 0.05). (D) Microscopic views of malignant mammary tumors; H&E staining of the cancer control, CIS+EPI, NCE, and FPNCE. Arrowhead, nuclear polymorphism; arrow, cancer tissue and cell mitosis (magnification, 400×; the scale bar represents 50 μm).

Animals were administered with CIS+EPI, NCE, and FPNCE for 20 days intraperitoneally or received no treatment for 20 days (the control group). The LD_50_ values of free EPI and CIS drugs were 8 and 10 mg/kg, respectively. The protocol employed for treating the mice is shown schematically in Figure 8A. At the end of the treatment period, the mouse weight and tumor volume were measured.

Weight changes in mice during the treatment period (Figure 8B(i),(ii)) and on the 20th day (Figure 8B(iii)) are presented. Also, changes in the tumor volume are observed during the treatment period (Figure 8C(i),(ii)) and on the last day of the treatment period (Figure 8C(iii)). The results showed that the group with FPNCE treatment had a low tumor growth rate. As shown in Figure 8D, the antitumor efficacy of combination therapy was enhanced by the FA-PEGylated niosomal system. In the cancer control, high mitosis and invasion, nuclear pleomorphism, and severe hyperchromicity were observed (score 3). A study of breast tissues treated with CIS+EPI showed a reduction in cancer cell invasion (score 2), while the rate of mitosis and nuclear polymorphism did not decrease compared to the cancer control group (score 3). In the NCE group, mitosis and invasion rates were reduced (score 2). In the FPNCE group, the mitosis and invasion (score 1) and nuclear pleomorphism were decreased (score 2) (Table 3 and Figure 8D).

**Table 3:** *In Vivo* Pathological Results

group	nuclear pleomorphism	mitosis index	invasion
cancer control	3	3	3
CIS+EPI	3	3	2
NCE	3	2	2
FPNCE	2	1	1

## 4 Discussion

Today, nanotechnology and medicine have combined to introduce a new stage of cancer therapy by increasing the effectiveness of chemotherapeutics and overcoming clinical challenges.^37−39^ In this study, a niosomal combination therapy of CIS and EPI was proposed to maintain high tumor termination while posing less of a threat to healthy organs. Various surface-modifying compounds (e.g., FA and PEG) were employed to augment the localization of drugs within tumor deposits and increase intracellular drug accumulation through enhanced endocytosis. PEG, a highly biocompatible and water-soluble polymer, was used for niosome surface modification.^41,43^ The functionalization of niosomes with PEG can increase the passive targeting of anticancer therapeutics and enhance the internalization of drugs into cancer cells.^44,45^ FA is one of the best active targeting ligands that bind to folate receptors with high affinity and internalize into cells through receptor-mediated endocytosis.^41,46^ Folate receptors are anchored cell surface receptors that are upregulated and overexpressed in numerous cancer cell types compared with normal cells.^47^

The results of drug release profiles related to each sample group showed that FPNCE revealed a pH-dependent manner in its release profile. This might be explained by the electrostatic interaction between the drugs and nonionic surfactants and the ionization state at physiological pH.^48^ pH-responsive delivery nanosystems can lead to the site-specific release of therapeutic cargos through cleaving of pH-sensitive bonds upon a pH gradient.^49^ According to our results, FPNCE and NCE enabled the codelivery of the drugs to the lower pH of 5.4. Particularly, the release of EPI and CIS from NCE and FPNCE formulations at the pH of 5.4 is notably higher than the release at the pH of 7.4 after 24, 48, and 72 h.

Interestingly, the dissociation of niosome particles in an acidic pH is related to the repulsion forces between several groups, including positively charged drugs and positively charged PEG chains.^50^ Furthermore, the FPNCE group exhibited a lower release rate than the NCE group in both conditions because high EEs and improved shelf-life of FA-PEGylated niosomal formulations prolonged the release profile during the process of drug delivery and the accumulation of the drug in tumor tissues. The sustained drug release signifies the advantage of nanoniosomal formulations in enhancing the antitumor effect and reducing systemic toxicity, compared to the free drugs.^51^ As our data suggested, the controlled release of the niosomal drugs presented high toxicity on 4T1 and SKBR3 cancer cells over 48 and 72 h while inducing low effects on the MCF10A healthy cell line.

In the present study, cellular effects of individual and combination therapy of CIS and EPI along with the drugs loaded into bare niosomes and PEG–FA-decorated niosomes were investigated. *In vitro* experiments were carried out against 4T1 and SKBR3 breast cancer cell lines. The FPNCE formulation showed a higher cytotoxic effect than other formulations and free drugs after 48 and 72 h incubation periods, which is perhaps due to folate receptor-mediated endocytosis that enhanced the cellular uptake of CIS and EPI.^52,53^ CIS-loaded niosomes were designed and studied for breast cancer treatment.^54,55^ It is also found that PEGylated liposomal CIS has a significantly amplified cytotoxicity effect on the human bladder carcinoma cell line after 24 and 48 h compared to the free CIS.^56^ Additionally, the survival rate of mouse malignant tumor cells treated with FA-conjugated CIS-PLGA nanoparticles was less than that of the group treated with free CIS.^57^

It is also worth noting that the minimum amount of folate-PEG derivatization was reported to allow for efficient recognition by the folate-binding protein.^58^ The high amount of free folate may otherwise avoid cellular uptake of niosomes through competitive binding to folate receptors on the cell surface. In addition, niosomes without drugs had no toxic effects on healthy cells.^59^ Our study proposed that FPNCE exhibits high cellular uptake, whereas nonfunctionalized niosomes failed to equally infiltrate into the cancer cells.

Scratch assays were performed to evaluate the inhibitory effects on tumor metastasis. According to the results, FPNCE showed excellent antimigratory effects in breast cancer cells (4T1 and SKBR3) after 72 h of treatment, while these effects were minimal with the use of drugs only. Furthermore, synergistic effects of CIS in combination with anticancer drugs such as gemcitabine and paclitaxel have been investigated against breast cancer and other diseases.^15,60,61^ Remarkably, as in our research, FPNCE yielded CIs lower than 1, which indicated synergism.

To take a closer look at the mechanism of apoptosis and determine the efficacy of niosomal formulations, the expression of eight different genes was measured. These genes could be divided into two groups: proapoptotic (*Bax*, *Caspase3*, *Caspase9*, and *Mfn1*) and antiapoptotic (*Bcl2*, *Drp1*, *MMP-2*, and *MMP-9*). FPNCE displayed better results in both increased expression of upregulating genes and decreased expression of downregulating ones in comparison to other groups. The data correspond well with the results of previous studies.^62−64^ In a study, it was reported that CIS-encapsulated liposomes induced apoptosis, activated *Caspase9* and *Caspase3*, downregulated *Bcl2*, and upregulated *Bax.*(65) In another study, it was shown that the EPI injection was indirectly linked with a lower expression of *MMP-2* and *MMP-9* genes; consequently, the adhesion, migration, and invasion properties of urothelial carcinoma cells had also been decreased.^66^

Based on the light microscopic study, the greatest histologic antitumor responses were seen in the FPNCE treatment group, with a low mitotic index and plenty of apoptotic cells. The tumor number was significantly decreased in tumor-bearing BALB/c mice receiving FPNCE compared to other groups. Intermediate nuclear pleomorphism and mild mitotic counts were seen in CIS+EPI. Similarly, there was an intermediate mitotic count and nuclear pleomorphism in the NCE treatment group. In the treatment with FPNCE, the antitumor effect occurred more by induction of apoptosis and inhibition of mitosis in tumor cells than NCE and CIS+EPI groups. The *in vivo* anticancer efficacy of FA-PEGylated niosomes also revealed that FPNCE caused a major tumor size reduction and body weight gain compared to the NCE and CIS+EPI groups. These findings were supported by another study where paclitaxel-loaded FA-PEGylated nanocrystals demonstrated higher antitumor efficacy than PEGylated nanocrystals, which in turn possessed higher efficacy than free paclitaxel.^67^

Our research introduced a nanoscale niosome for the codelivery of CIS and EPI drugs. While several studies have suggested them as potential chemotherapeutics in cancer therapy, their combination has suffered from systemic toxicity and serious side effects.^68^ Previous works have investigated the effects of CIS and EPI loaded in different nanosystems on a wide range of cancer cells; however, there has been a gap in studying their combination through a delivery system and investigating the synergism.^53,69^ Using CIS and EPI as free drugs and their combination for cancer treatment requires their high dosages during the treatment period, which eventually escalates the side effects and augments toxicity effects toward normal cells.^69^ Our results indicated that using biocompatible niosomes modified with FA and PEG amplifies the antitumor activity of CIS and EPI at low concentrations by improving apoptosis and endocytosis in 4T1 and SKBR3 breast cancer cells and reducing migration and invasion rates in BALB/c mice. The local delivery of drugs, cytotoxicity efficacy, and the creation of apoptotic bodies indicated that the FA-PEGylated nanoniosome serves as a suitable nanocarrier for dual delivery of the drugs.

## 5 Conclusions

In this study, FA-PEGylated niosome-based nanocarriers were successfully fabricated for the codelivery of CIS and EPI. The obtained functionalized nanoscale niosomes exhibited enhanced stability during 2 months and sustained release in physiological pH. The cellular results also demonstrated that the FPNCE group revealed antitumor activity toward SKBR3 and 4T1 cancer cells and exhibited lower cytotoxicity toward healthy cells. Moreover, the inhibited migration and division of cancer cells were greater for the FPNCE and NCE groups compared to free drugs. In general, the proposed functionalized niosomal nanocarrier could be a promising approach for breast cancer treatment.

## Supporting Information Available

Chemicals and characterization, preparation of different niosomal formulations, primer sequences in real-time PCR, and kinetic release models and parameters (PDF)
