# Supplementary material for: pH-Responsive, Adorned Nanoniosomes for Codelivery of Cisplatin and Epirubicin: Synergistic Treatment of Breast Cancer
Source: ACS Appl Bio Mater. 2022 Feb 7;5(2):675–90. doi: 10.1021/acsabm.1c01107 (PMC8864616; doi:10.1021/acsabm.1c01107)
Supplement: Supplementary file 1 — mt1c01107_si_001.pdf [file mt1c01107_si_001.pdf]

Supporting Information

pH-Responsive, Adorned Nanoniosomes for Co-delivery of Cisplatin and Epirubicin: Synergistic Treatment of Tumorigenesis Breast Cancer

Ali Moammeri[1,†], Koorosh Abbaspour[1,†], Alireza Zafarian[2,†], Elham Jamshidifar[3,†], Hamidreza Motasadizadeh[3,4], Farnaz Dabbagh Moghaddam[5], Zeinab Salehi[1,*], Pooyan Makvandi[6,*], Rassoul Dinarvand[3,4,*]

[1]School of Chemical Engineering, College of Engineering, University of Tehran, Tehran, 111554563, Iran

[2]Faculty of Medicine, Isfahan University of Medical Sciences, Isfahan, 8174673461, Iran

[3]Department of Pharmaceutical Nanotechnology, Faculty of Pharmacy, Tehran University of Medical Sciences, Tehran, 141556451, Iran

[4]Nanotechnology Research Center, Faculty of Pharmacy, Tehran University of Medical Sciences, Tehran, 1316943551, Iran

[5]Department of Biology, Science and Research Branch, Islamic Azad University, Tehran, 1477893855, Iran

[6]Istituto Italiano di Tecnologia, Centre for Materials Interface, Pontedera 56025, Pisa, Italy

***Corresponding authors**

E-mail: zsalehy@ut.ac.ir (Z. Salehi); pooyan.makvandi@iit.it (P. Makvandi); Dinarvand@tums.ac.ir (R. Dinarvand)

1. Materials and Methods

1.1. Chemicals

EPI, CIS, and DOX were granted by the Tofigh-Darou Research & Engineering Company (Iran). Cholesterol, Span® 20, Span® 60, Span® 80, sodium dodecyl sulfate (SDS), and dialysis membrane (MWCO 12 kDa) were purchased from Sigma Aldrich (USA). Chloroform, Amicon (Ultra15-Membrane, MWCO 30kDa), FA-PEG-2000, and dimethyl sulfoxide (DMSO) were purchased from Merck (Germany). Medium RPMI-1640, DMEM (Dulbecco's Adjusted Eagle Medium), trypsin-EDTA, trypan blue, fetal bovine serum (FBS), phosphate-buffered saline (PBS), MTT (dimethylthiazol-2-yl-)-2,5, and penicillin/streptomycin (PS)100 X were obtained from Gibco, (USA). The Pasteur Institute, Iran, provided the 4T1 murine mammary carcinoma and SKBR3 human mammary carcinoma. Annexin V-FITC Flow Cytometry kit was obtained from Affymetrix biosciences (USA). Transgene Biotech, China (Cat No. ER101-01 and AE301-02), provided RNA extraction and cDNA synthesis kits.

1.2. Characterization

The structure and features of all formulations were examined by Fourier transform infrared (FT-IR) to determine and analyze the interaction between the different components (Hitachi, Japan 295 spectrophotometer) using the KBr disc method. The samples were examined in the range of 400-4000 cm^{-1}. Finally, samples were read by scanning with PerkinElmer Spectrum Software (Model 1600, Massachusetts, US). Malvern zeta sizer (Malvern Instrument Ltd. Malvern, UK) was applied to distribute the size and PDI based on dynamic light scattering (DLS). To investigate the optimum formulation morphology, transmission electron microscopy (TEM) at 80 KV (Netherland, Philips CM30) and scanning electron microscopy (SEM) (SSX-500, Shimadzu, Japan) were applied.

2. Results and Discussion

According to the results, the particle size correlates to the cholesterol-to-surfactant ratio (**Table S1**). The synthesized niosomes showed increased particle size at higher cholesterol-to-surfactant ratio and elevated lipid amount. The addition of cholesterol to niosome formulations increased the particle size by enhancing the rigidity of plasma membranes and the resistance post-sonication, resulting in the generation of larger-sized niosomes [1]. Low concentrations of cholesterol made the vesicular membranes more susceptible to being affected by ultrasound waves, giving rise to smaller-sized particles [2, 3]. As the results indicate, the values of EE for each drug increased with the addition of cholesterol, because the lipid layer on the niosome

surface got thicker, which contributed to the stability of niosomes and reduced their permeability to solutes [4]. Moreover, the results showed that even though Span® 80 gave higher EE and lower particle size, Span® 60 demonstrated better homogeneity than other surfactants in niosomal formulation, resulting in lower PDI. Niosomes containing Span® 80 had a smaller size than Span® 60, because Span® 80 has a higher hydrophobicity than Span® 60, which decreases surface free energy and particle size [5]. In addition, the results disclosed that niosomes containing Span® 20 had a larger average diameter than those with Span® 60. This might be due to the fact that Span® 20 has a higher hydrophilic-lipophilic balance (HLB) compared with Span® 60. In fact, a higher HLB value of surfactants leads to greater size of niosomes. Moreover, niosomes containing Span® 60 had less permeable membrane, which increased the entrapment of loaded drugs [6]. Even with these adjustments, niosomes should be equipped with several groups to ensure longevity in blood circulation and retention of the drug in target cells [7]. According to the results, the size of drug-loaded niosomes was not significantly affected by PEG modification. Subsequently, improving stability, increasing drug encapsulation, and reduced drug release are due to the PEGylation in the niosomal formulations. The increase in EE could be explained by the strong affinity of PEG with the non-ionic surfactants [8, 9]. On the other hand, the increase in EE could be attributed to the ability of PEG to block the leaking space in the vesicular membranes [10]. The average diameter of FPNCE was larger than pure niosomes, because FA-PEG-2000 increased the tension of the bilayer membrane on the niosome surface [11]. However, the PDI of FPNCE was reduced compared to the pure niosome, due to the electrostatic repulsion after functionalization with FA [12]. In addition, the variation in particle size, PDI, and EE over 60 days demonstrated that the FPNCE group was more stable than non-functionalized niosomes. The results of stability revealed that modification of niosome surface with PEG minimizes the problems associated with niosomal instability, such as drug leakage, fusion, and aggregation [10].

Table S1. Effect of the surfactant:cholesterol with various molar ratios, surfactant type, and lipid content on EE, LE, size, and PDI for different formulations

Systems	Formulations	Surfactant: Cholesterol (Molar ratio)	Lipid (µmol)	Surfactant Type	Drug Content (mg)	Vesicle size (nm)	PDI	EE (%), CIS	EE (%), EPI	LE (%), CIS	LE (%), EPI

**NCE**	NCE1	1:1	200	Span® 20	10/10	250.1	0.219	73.43	50.19	10.71	10.71
	NCE2	1:1	200	Span® 60	10/10	170.3	0.145	82.91	61.75	9.83	9.83
	NCE3	1:1	200	Span® 80	10/10	165.3	0.175	84.29	63.12	9.84	9.84
	NCE4	1:2	300	Span® 20	10/10	279.4	0.159	76.10	62.96	7.81	7.81
	NCE5	1:2	300	Span® 60	10/10	184.0	0.103	85.48	68.52	6.90	6.90
	NCE6	1:2	300	Span® 80	10/10	221.7	0.184	87.92	69.25	6.92	6.92
**FPNCE**	FPNCE	1:2	300	Span® 60	10/10	192.5	0.142	91.24	71.93	6.90	6.90
**Niosome**	N	1:2	300	Span® 60	-	158.3	0.032	-	-	-	-

Table S2. The used primer sequences in the Real-Time PCR

Genes	Forward primer (5'-3')	Reverse primer (3'-5')
Mfn1	GTTTTAGTAGACAGCCCAG	GTCCGTGTTCATCAGTGTT
Drp1	TTTGCTAGATGTGCCAGTTCC	ATTACTGCCTTTGGGACACTG
Bax	CGGCAACTTCAACTGGGG	TCCAGCCCAACAGCCG
Bcl2	GGTGCCGGTTCAGGTACTCA	TTGTGGCCTTCTTTGAGTTCG
Caspase3	CATACTCCACAGCACCTGGTTA	ACTCAAATTCTGTTGCCACCTT
Caspase9	CATATGATCGAGGACATCCAG	TTAGTTCGCAGAAACGAAGC
MMP-2	TTG ACG GTA AGG ACGGAC TC	CAT ACT TCA CAC GGA CCA CTTG
MMP-9	GCACGACGTCTTCCAGTACC	CAGGATGTCATAGGTCACGTAGC
ß-actin	TCCTCCTGAGCGCAAGTAC	CCTGCTTGCTGATCCACATCT

Table S3. Kinetic release models and parameters obtained for optimum niosomal formulation (a) for CIS and (b) for EPI

**(a) Release Model**	Equation	R^2				
		NCE-CIS (pH=7.4-37°C)	NCE-CIS (pH=5.4-37°C)	FPNCE - CIS (pH=7.4-37°C)	FPNCE - CIS (pH=5.4-37°C)	CIS solution (pH=7.4-37°C)

Zero-Order	$C_t=C_0+K_0t$	R^2=0.8560	R^2=0.7409	R^2=0.8678	R^2=0.7461	R^2=0.4767
Korsmeyer-Peppas	$M_t/M_\infty=Kt^n$	R^2=0.9649	R^2=0.9161	R^2=0.9674	R^2=0.8749	R^2=0.7858
		n=0.5104	n=0.4514	n=0.5232	n=0.4264	n=0.4028
First-Order	LogC=Log C_0+Kt/2.30	R^2=0.8986	R^2=0.8542	R^2=0.8974	R^2=0.8232	R^2=0.9056
Higuchi	$Q=KH\sqrt{t}$	R^2=0.9627	R^2=0.8862	R^2=0.9684	R^2=0.8770	R^2=0.6488

**(b) Release Model**	Equation	R^2				
		NCE-EPI (pH=7.4-37°C)	NCE-EPI (pH=5.4-37°C)	FPNCE - EPI (pH=7.4-37°C)	FPNCE - EPI (pH=5.4-37°C)	EPI solution (pH=7.4-37°C)
Zero-Order	$C_t=C_0+K_0t$	R^2=0.9086	R^2=0.9040	R^2=0.9253	R^2=0.9031	R^2=0.3997
Korsmeyer-Peppas	$M_t/M_\infty=Kt^n$	R^2=0.9931	R^2=0.9850	R^2=0.9964	R^2=0.9950	R^2=0.7250
		n=0.4943	n=0.4353	n=0.5124	n=0.4667	n=0.3578
First-Order	LogC=Log C_0+Kt/2.30	R^2=0.9492	R^2=0.9617	R^2=0.9567	R^2=0.9451	R^2=0.8232
Higuchi	$Q=KH\sqrt{t}$	R^2=0.9838	R^2=0.9809	R^2=0.9888	R^2=0.9812	R^2=0.5639

References

1. Barani, M.; Nematollahi, M. H.; Zaboli, M.; Mirzaei, M.; Torkzadeh-Mahani, M.; Pardakhty, A.; Karam, G. A., In silico and in vitro study of magnetic niosomes for gene delivery: The effect of ergosterol and cholesterol. *Mater. Sci. Eng. C* **2019,** *94*, 234-246.
2. Khan, M. I.; Madni, A.; Hirvonen, J.; Peltonen, L., Ultrasonic processing technique as a green preparation approach for diacerein-loaded niosomes. *AAPS PharmSciTech* **2017,** *18* (5), 1554-1563.
3. Jadhav, C.; Kate, V.; Payghan, S. A., Investigation of effect of non-ionic surfactant on preparation of griseofulvin non-aqueous nanoemulsion. *J NANOSTRUCTURE CHEM* **2015,** *5* (1), 107-113.
4. Nematollahi, M. H.; Pardakhty, A.; Torkzadeh-Mahanai, M.; Mehrabani, M.; Asadikaram, G., Changes in physical and chemical properties of niosome membrane induced by cholesterol: a promising approach for niosome bilayer intervention. *RSC Adv.* **2017,** *7* (78), 49463-49472.
5. Foo, K. S.; Bavoh, C. B.; Lal, B.; Mohd Shariff, A., Rheology impact of various hydrophilic-hydrophobic balance (HLB) index non-ionic surfactants on cyclopentane hydrates. *Molecules* **2020,** *25* (16), 3725.
6. Bnyan, R.; Khan, I.; Ehtezazi, T.; Saleem, I.; Gordon, S.; O'Neill, F.; Roberts, M., Surfactant effects on lipid-based vesicles properties. *J Pharm Sci* **2018,** *107* (5), 1237-1246.
7. Tavano, L.; Muzzalupo, R., Multi-functional vesicles for cancer therapy: the ultimate magic bullet. *Colloids Surf. B* **2016,** *147*, 161-171.
8. Ge, X.; Wei, M.; He, S.; Yuan, W.-E., Advances of non-ionic surfactant vesicles (niosomes) and their application in drug delivery. *Pharmaceutics* **2019,** *11* (2), 55.
9. Maleki Dizaj, S., Preparation and study of vitamin A palmitate microemulsion drug delivery system and investigation of co-surfactant effect. *J NANOSTRUCTURE CHEM* **2013,** *3* (1), 59.

10. Yu, J.; Mao, J.; Nagao, M.; Bu, W.; Lin, B.; Hong, K.; Jiang, Z.; Liu, Y.; Qian, S.; Tirrell, M., Structure and dynamics of lipid membranes interacting with antivirulence end-phosphorylated polyethylene glycol block copolymers. *Soft matter* **2020,** *16* (4), 983-989.
11. Garni, M.; Thamboo, S.; Schoenenberger, C.-A.; Palivan, C. G., Biopores/membrane proteins in synthetic polymer membranes. *Biochim Biophys Acta Biomembr* **2017,** *1859* (4), 619-638.
12. Brandt, J. V.; Piazza, R. D.; Dos Santos, C. C.; Vega-Chacón, J.; Amantéa, B. E.; Pinto, G. C.; Magnani, M.; Piva, H. L.; Tedesco, A. C.; Primo, F. L., Synthesis and colloidal characterization of folic acid-modified PEG-b-PCL Micelles for methotrexate delivery. *Colloids Surf. B* **2019,** *177*, 228-234.
